# Rapid paracellular transmigration of *Campylobacter jejuni* across polarized epithelial cells without affecting TER: role of proteolytic-active HtrA cleaving E-cadherin but not fibronectin

**DOI:** 10.1186/1757-4749-4-3

**Published:** 2012-04-25

**Authors:** Manja Boehm, Benjamin Hoy, Manfred Rohde, Nicole Tegtmeyer, Kristoffer T Bæk, Omar A Oyarzabal, Lone Brøndsted, Silja Wessler, Steffen Backert

**Affiliations:** 1 From the School for Medicine and Medical Science, University College Dublin, Belfield Campus, Dublin-4, Ireland; 2 The Division of Microbiology, University Salzburg, A-5020, Salzburg, Austria; 3 The Department of Medical Microbiology, Helmholtz Center for Infection Research, Inhoffen Str. 7, D-38124, Braunschweig, Germany; 4 The Department of Veterinary Disease Biology, University Copenhagen, Stigbøjlen 4, DK-1870, Frederiksberg C, Denmark; 5 Institute for Environmental Health, Inc., 15300 Bothell Way NE Lake Forest Park, Seattle, WA, 98155, USA; 6 University College Dublin, UCD School of Biomolecular and Biomedical Sciences, Science Center West L231, Belfield Campus, Dublin 4, Ireland

**Keywords:** HtrA, E-cadherin, Fibronectin, MKN-28, Molecular pathogenesis, Cellular invasion, Signaling, TER, Virulence

## Abstract

**Background:**

*Campylobacter jejuni* is one of the most important bacterial pathogens causing food-borne illness worldwide. Crossing the intestinal epithelial barrier and host cell entry by *C. jejuni* is considered the primary reason of damage to the intestinal tissue, but the molecular mechanisms as well as major bacterial and host cell factors involved in this process are still widely unclear.

**Results:**

In the present study, we characterized the serine protease HtrA (high-temperature requirement A) of *C. jejuni* as a secreted virulence factor with important proteolytic functions. Infection studies and in vitro cleavage assays showed that *C. jejuni*’s HtrA triggers shedding of the extracellular E-cadherin NTF domain (90 kDa) of non-polarised INT-407 and polarized MKN-28 epithelial cells, but fibronectin was not cleaved as seen for *H. pylori*’s HtrA. Deletion of the *htrA* gene in *C. jejuni* or expression of a protease-deficient S197A point mutant did not lead to loss of flagella or reduced bacterial motility, but led to severe defects in E-cadherin cleavage and transmigration of the bacteria across polarized MKN-28 cell layers. Unlike other highly invasive pathogens, transmigration across polarized cells by wild-type *C. jejuni* is highly efficient and is achieved within a few minutes of infection. Interestingly, E-cadherin cleavage by *C. jejuni* occurs in a limited fashion and transmigration required the intact flagella as well as HtrA protease activity, but does not reduce transepithelial electrical resistance (TER) as seen with *Salmonella, Shigella*, *Listeria* or *Neisseria.*

**Conclusion:**

These results suggest that HtrA-mediated E-cadherin cleavage is involved in rapid crossing of the epithelial barrier by *C. jejuni* via a very specific mechanism using the paracellular route to reach basolateral surfaces, but does not cleave the fibronectin receptor which is necessary for cell entry.

## Introduction

Infections with pathogenic food-borne bacteria constitute one of the leading causes of morbidity and mortality in humans. The World Health Organization (WHO) suggests that the human population worldwide suffers from about 4.5 billion incidences of gastroenteritis annually, causing approximately 1.8 million deaths [1]. Various *Campylobacter* species have been identified as the leading enteric bacterial infection worldwide [2,3]. *Campylobacter jejuni* is considered as a classical zoonotic pathogen, as it is found in the normal intestinal flora in many birds and mammals. Since *C. jejuni* colonizes various food animals, it can contaminate food products during processing [4]. After ingestion by a human host, these bacteria use their flagella-driven motility to colonize the epithelial cells of the ileum and colon. Here, they can interfere with normal functions in the intestinal tract, leading to diseases associated with fever, malaise, abdominal pain and watery diarrhoea [2,3]. In addition, a minority of infected individuals may develop late complications such as Reiter’s reactive arthritis or Guillain-Barrè and Miller-Fisher syndromes [5]. There is increasing evidence showing that *C. jejuni* disturbs the normal absorptive capacity of the human intestine by damaging epithelial cell functions, either by cell invasion, the production of pathogenicity-associated factors or indirectly by triggering inflammatory responses [3,6-8].

It has been proposed that transmigration across and invasion into intestinal epithelial cells during infection is a major reason of *C. jejuni*-triggered tissue damage [2-4]. Investigation of gut biopsies obtained from infected patients and in vitro infection experiments of intestinal epithelial cells indicated that *C*. *jejuni* can enter human host cells [9-11]. *Campylobacter jejuni* expresses various adhesins in the outer-membrane including CadF, FlpA, JlpA and PEB1 [12-15]. For example, in vitro CadF is a well-known bacterial factor that binds to fibronectin, an important extracellular matrix (ECM) protein and bridging factor to integrin receptors [13,16,17]. Maximal bacterial adherence and invasion of INT-407 intestinal epithelial cells is dependent on CadF and is associated with tyrosine phosphorylation of paxillin, a focal adhesion-based scaffolding factor [18]. The expression of CadF also seems to be required for the stimulation of the small Rho GTPases Rac1 and Cdc42 via fibronectin and integrin member β1, that are required for *C. jejuni* host cell entry. The signalling pathways involved in the latter process have been described in detail [19-21]. However, fibronectin and integrin β1 are basolateral receptor molecules and not commonly exposed at apical surfaces in the intestine. It is therefore unclear how *C. jejuni* gains access to these receptors during infection.

To access deeper tissues and cause short- or long-term infections in the human body, various pathogenic bacteria, including *Salmonella, ShigellaListeria* or *Neisseria*, must overcome the epithelial barrier [22,23]. These important bacterial pathogens are able to cross polarised intestinal epithelial cells by different mechanisms, known as the paracellular and the transcellular routes. Bacteria using the transcellular route enter host cells at apical surfaces followed by intracellular trafficking and leave these cells at the basolateral surface. In contrast, bacteria specialised on the paracellular route cross the epithelial barrier by passage between neighbouring epithelial cells and overcome the tight junctions and adherens junctions [24]. In the case of *C. jejuni*, the literature is highly controversial. While some groups reported the paracellular route, others described the transcellular model or a mix of both [25-30]. In general, the host factors and bacterial factors involved in the transmigration process of *C. jejuni* are still unclear [31].

We have recently shown that a closely related pathogen, *Helicobacter pylori,* secretes a novel bacterial virulence determinant into the culture supernatant, the serine protease HtrA [32-34], which is also present in *C. jejuni*[35-37]. HtrA proteins constitute a group of heat shock induced serine proteases that influence the adhesion and invasion properties of different bacterial pathogens. HtrA proteins typically consist of a signal peptide, a trypsin-like serine protease domain and one or two protein interaction (PDZ) domains. In addition, by binding of the PDZ domain in one HtrA molecule to that in other HtrA molecules, HtrA can build-up to highly proteolytic active oligomers that also function as a chaperone [38]. The HtrA protease domain consists of an active site, called the catalytic triad, which is formed by the conserved amino acid residues histidine, aspartatic acid and serine [39]. Many bacterial HtrA proteins are suggested to be localized in the periplasm and to be involved in quality control of envelope proteins by degradation of misfolded proteins as well as prevention of formation of aggregates [40]. Thus, it was surprising to find that HtrA exhibits the capability of extracelluar transport in *H. pylori*[34,41], where it could cleave host surface molecules. We identified that *H. pylori* HtrA directly cleaves the junctional protein and tumor suppressor E-cadherin and fibronectin on the surface of gastric epithelial host cells. HtrA-mediated cleavage of E-cadherin facilitated the loss of the adherence junction complex leading to the disruption of the epithelial barrier function in response to *H. pylori* infection [32] and may also apply to *C. jejuni* HtrA [33]. Here, we present the results from a detailed investigation to determine if *C. jejuni* HtrA can cleave both E-cadherin and fibronectin, and whether HtrA protease activity is required for transmigration across polarised epithelial cells. Our findings show that *C. jejuni* can effectively cross polarised epithelial cells in an HtrA-protease dependent fashion without affecting TER.

## Results & discussion

### HtrA protease is conserved in *H. pylori* and *C. jejuni*

Recently, HtrA of the gastric pathogen *H. pylori* was reported to be specifically secreted into the cell culture supernatant, where it can cleave the ectodomain of the host cell adhesion protein and tumour-suppressor E-cadherin, and degrades fibronectin [32]. A sequence alignment of HtrAs from different *C. jejuni* and *H. pylori* strains was performed and revealed a high degree of similarity between the HtrA domains and protein sequences (Additional file 1: Figure S1A). We also found that the amino acids in the catalytic triad (histidine, aspartate and serine) are conserved and at the expected position among these proteins ( Additional file 1: Figure S1B, shaded with yellow). These results suggest that HtrA’s are highly conserved in various *C. jejuni* isolates. We therefore suspected that *C. jejuni* may also use its HtrA protease to cross the barrier of polarised epithelial cells.

### Analysis of wild-type and *htrA* mutant *C. jejuni* by electron microscopy and motility assays

**Figure 1:**
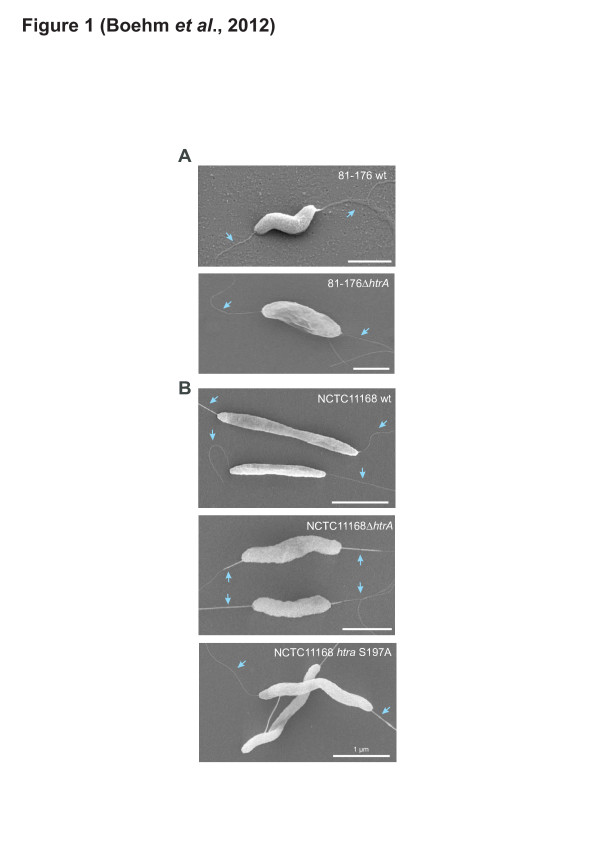
**Analysis of*C. jejuni*and*htrA*mutants by scanning electron microscopy and motility assays.** Scanning electron microscopy revealed that wild-type (wt) *C. jejuni* and different *htrA* mutants of strain 81–176 **(A)** and NCTC11168 **(B)** produce intact bipolar flagella (blue arrows) and only slight morphological differences. Each bar corresponds to 1 μm

**Figure 2:**
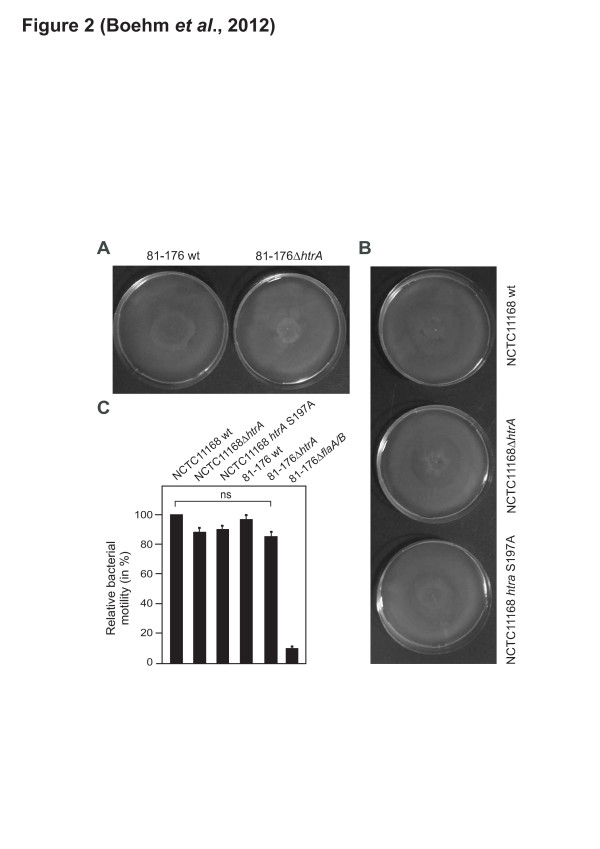
**Different*C. jejuni*wild-type and*htrA*mutant strains exhibit high motility.** Motility assays were performed for 36 h on agar using wild-type (wt) *C. jejuni* and different *htrA* mutants of strain 81–176 **(A)** and NCTC11168 **(B)** and revealed high motility for each strain. **(C)** Quantification of motility using the indicated wt and mutant *C. jejuni* strains. One hundred percent of motility corresponds to the highest swarming capability presented by *C. jejuni* wt strain NCTC11168. No significant (ns) differences were seen among the various highly motile strains. The Δ*flaA/B* mutant was used motility-deficient a control

We first aimed to investigate several *htrA* mutants in *C. jejuni*, including the wild-type (wt) strains 81–176 and NCTC11168, their isogenic Δ*htrA* deletion mutants and NCTC11168 *htrA* S197A, a strain with complemented *htrA* carrying a point mutation at S197A in the active centre, rendering the protein catalytically inactive [36]. The morphology of the produced *C. jejuni* strains was analysed by scanning electron microscopy (FESEM). Comparison of the *C. jejuni* wt strains 81–176 and NCTC11168 with their corresponding *htrA* mutants revealed some slight differences at the bacterial surface, but no major phenoptypical alterations were noted (Figure 1). In addition, all *htrA* mutants produced intact bipolar flagella as compared to their wt counterparts (Figure 1, blue arrows). Studies of all strains by another electron microscopic method (negative staining) revealed similar results and thus confirmed our findings (Additional file 1: Figure S2). Motility assays revealed that both *C. jejuni* wt and *htrA* mutant strains were highly motile, suggesting that mutation of *htrA* does not significantly affect this important pathogenicity property of the bacteria (Figure 2).

### HtrA’s of strains 81–176 and NCTC11168 form active multimers

**Figure 3:**
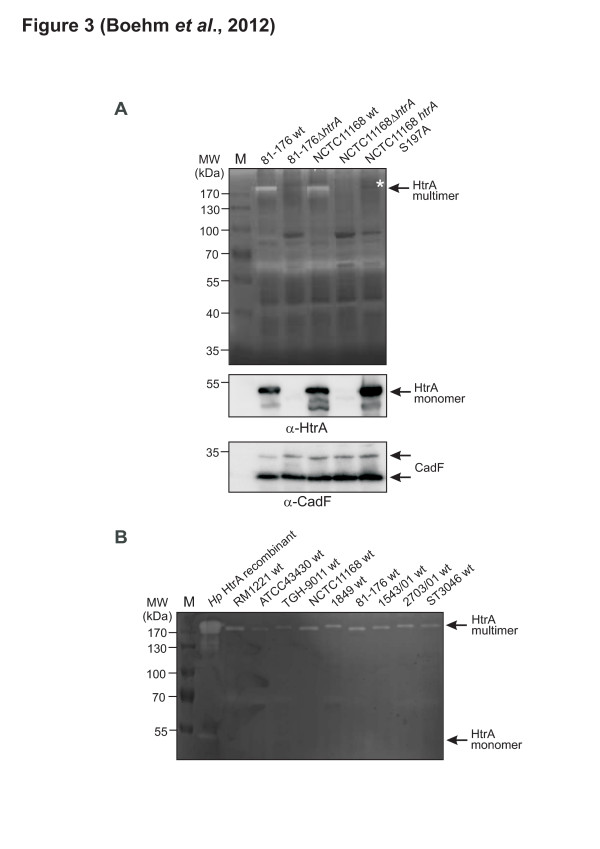
**Detection of proteolytic active HtrA multimers in different*C. jejuni*wild-type and mutant strains. (A)** Total cell lysates of plate-grown *C. jejuni* 81–176 wild-type (wt), 81-176Δ*htrA,* NCTC11168 wt, NCTC11168Δ*htrA* and NCTC11168 *htrA* S197A were investigated for protease activities by casein zymography. The position of active monomeric and multimeric HtrA proteins is indicated with arrows. Active monomeric HtrA was only weakly visible as indicated. The asterisk indicates the inactive HtrA multimer formed by the S197A point mutant. **(B)** Total cell lysates of the indicated plate-grown wt strains were separated on non-denaturing gels. Proteolytic active HtrA bands were detected by casein zymography as described. Purified recombinant *H. pylori* (*Hp*) HtrA served as control

We tested the assumption that *C. jejuni* wt strains 81–176 and NCTC11168 can generate proteolytic active HtrA multimers. For this purpose, the wt strains and corresponding isogenic *ΔhtrA* deletion and S197A mutants were grown in BHI broth medium, followed by casein zymography of total cell lysates [32]. The results show that the HtrA protein was not synthesized by the *ΔhtrA* mutants, whereas HtrA is produced by the wt and S197A isolates, and only wt *C. jejuni* formed caseinolytic active multimers as expected (Figure 3A). These observations are in agreement with reports on HtrA in other bacteria such as *E. coli* where the HtrA multimers are highly proteolytic active rather than the monomer [38]. The identity of *C. jejuni* HtrA monomers and multimers was approved by mass spectrometry of the excised bands as described [33]. The presence or absence of HtrA expression was further confirmed by anti-HtrA Western blotting using an anti-CadF blot as loading control (Figure 3A, bottom).

### Multiple *C. jejuni* wt strains express active HtrA multimers

To exclude the possibility that HtrA activity is restricted to the above *C. jejuni* isolates, we tested a larger collection of wt strains for their expression of active HtrA proteins. Total cell lysates from *C. jejuni* wt RM1221, ATCC43430, TGH-9011, 1849, 1543/01, 2703/01 and ST3046 were prepared and analyzed for HtrA protease activities by casein zymography. All tested strains expressed the active HtrA multimer with a molecular size of ~200 kDa, albeit at various extent, while only faint bands of the monomer at ~53 kDa were seen (Figure 3B). We could also confirm previous findings that these native *C. jejuni* HtrA’s were very similar to recombinant *H. pylori* HtrA forming highly active multimers at the same size ~200 kDa [33] (Figure 3B, lane 1).

### *C. jejuni* secretes HtrA into the culture supernatant where they form active multimers

**Figure 4:**
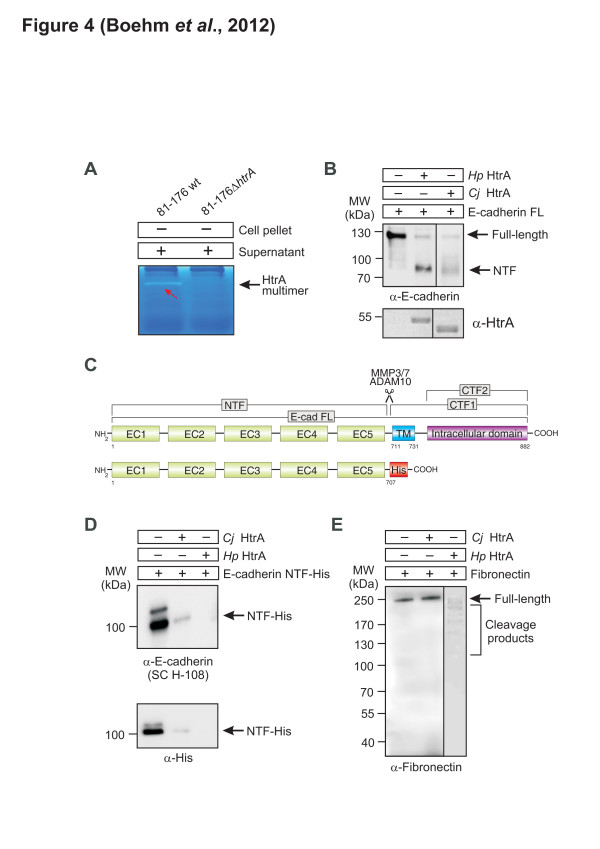
***C. jejuni*HtrA is secreted into the supernatant, forms proteolytic active multimers and recombinant*C. jejuni*HtrA cleaves E-cadherin but not fibronectin. (A)** Filtered supernatants of broth-grown wild-type (wt) or *htrA* mutant *C. jejuni* (*Cj*) were subjected to zymography to detect proteolytic active HtrA multimers. **(B)** In vitro cleavage of full-length (FL) E-cadherin incubated with purified *Cj* HtrA or *H. pylori* (*Hp*) HtrA results in shedding of the extracellular N-terminal fragment (NTF). The anti-HtrA blot serves as loading control. **(C)** Schematic presentation of the domain structure of full-length human E-cadherin (GenBank accession number CAA78353.1) and the His-tagged NTF domain used for cleavage assays. E-cadherin consists of five extracellular domains (EC1-EC5) comprising the NTF domain, a transmembrane (TM) domain and an intracellular domain called C-terminal fragment (CTF). The cleavage site by some host cell proteases is indicated. **(D)** In vitro cleavage of the recombinant E-cadherin NTF domain incubated with purified *Cj* HtrA or *Hp* HtrA results in cleavage and disappearance of the 90 kDa NTF fragment. **(E)** In vitro cleavage assay of fibronectin incubated with purified HtrAs under identical conditions shows that fibronectin can be cleaved by *Hp* HtrA but not *Cj* HtrA. All reactions were incubated for 16 h at 37°C.

The remarkable sequence homology between HtrA’s from *H. pylori* and *C. jejuni* led us to propose that active *C. jejuni* HtrA maybe also secreted into the cell culture supernatant by various strains, similar to its *H. pylori* counterpart. To test this hypothesis, *C. jejuni* wt strains 81–176 and NCTC11168 and its isogenic *ΔhtrA* deletion mutants were grown in BHI broth medium and bacteria-free supernatants were prepared, followed by casein zymography. The results show that the HtrA proteins from wt *C. jejuni* were found in the bacterial culture supernatant fraction where they also form caseinolytic active multimers (Figure 4A and data not shown).

### In vitro cleavage properties of purified HtrA’s of *C. jejuni* and *H. pylori*

*H. pylori* HtrA has recently been shown to cleave the cell adhesion protein E-cadherin and the extracellular matrix protein fibronectin [32]. Full length E-cadherin has a molecular weight of about 130 kDa and is composed of a ~90 kDa extracellular domain amino-terminal fragment (NTF) and a ~40 kDa carboxy-terminal fragment (CTF1) [32]. To determine whether *C. jejuni* HtrA can cleave E-cadherin into specific subfragments recombinant HtrAs was purified. As expected, *C. jejuni* HtrA had slightly different molecular weight as compared to its *H. pylori* counterpart, due to the smaller size of the expressed protein (472 vs. 476 amino acids) (Additional file 1: Figure S3). Recombinant *C. jejuni* HtrA was then incubated with recombinant full-length E-cadherin. The ectodomain shedding of E-cadherin was detected using α-E-cadherin antibodies recognising the EC5 subunit in the NTF domain. Like its *H. pylori* counterpart, it was shown that *C. jejuni* HtrA cleaved E-cadherin as monitored by the disappearance of full-length protein band and increasing amounts of the ~90 kDa NTF domain (Figure 4B /C). In addition, we performed assays with the purified recombinant NTF domain (amino acids 1–707 followed by a His-tag) showing that this domain disappears upon HtrA cleavage as shown by anti-E-cadherin and anti-His blots, indicating that the cleavage site of HtrA is indeed in the extracellular part of E-cadherin adjacent to the transmembrane domain (amino acids 711–731) (Figure 4C /D). Interestingly, E-cadherin ectodomain cleavage by *C. jejuni* HtrA was not as efficient as compared to *H. pylori* HtrA, but band sizes were similar, albeit with different intensity. Moreover, *H. pylori* HtrA cleaved purified fibronectin into multiple subfragments, while *C. jejuni* HtrA did not cleave fibronectin at all (Figure 4E). These observations suggest that although HtrA from *H. pylori* and *C. jejuni* share substantial sequence homology, significant differences exist for certain host substrates. The above experiments were all performed at 37°C, which is the body temperature of mammalian hosts. However, since *C. jejuni* also exhibits host specificity for avian species (42°C), we tested if HtrA exhibits different cleavage properties at 42°C. Interestingly, the cleavage patterns were identical between 37°C and 42°C (Figure 4C /D and Additional file 1: Figure S4).

### In vivo cleavage of E-cadherin in *C. jejuni* infected INT-407 or MKN-28 cells

**Figure 5:**
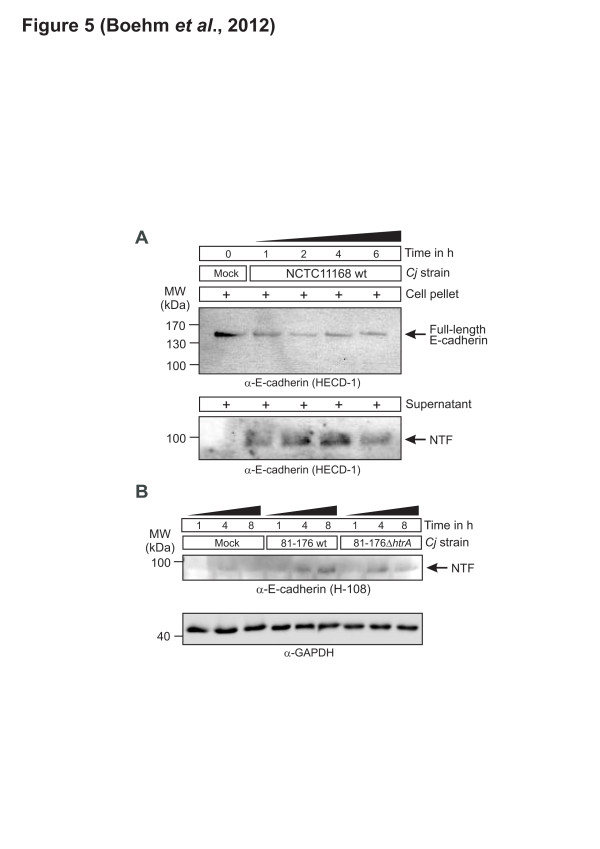
**In vivo cleavage of E-cadherin during*C. jejuni*infection of non-polarised INT-407 cells and polarised MKN-28 cells. (A)** INT-407 cells were infected with *C. jejuni* NCTC11168 wild-type (wt) strain in a time course. The Western blot shows changes of cell-associated full-length E-cadherin and reveals the generation of 90 kDa NTF domain in the supernatant over time. **(B)** Polarised MKN-28 cells were infected with *C. jejuni* 81–176 wt and *htrA* mutant strains in a time course. Supernatants were harvested at each time point, and the E-cadherin NTF fragment was detected using the indicated antibody. GAPDH expression levels were determined as loading control of total cellular protein

The next aim was to investigate if *C. jejuni* HtrA can cleave E-cadherin during infection in vivo. For this purpose, E-cadherin-expressing INT-407 and MKN-28 cells were infected with *C. jejuni* wt strains NCT11168 or 81–176 in a time course for the indicated periods of time (up to 8 h) and the cleavage of E-cadherin was determined by immunoblotting. The results show that the overall amount of full-length E-cadherin dropped down during infection, but was not eliminated (Figure 5A). In addition, the signals of the entire 90 kDa NTF increased over time up to 4 hours and then dropped somewhat, as detected in the supernatant of infected cells (Figure 5B). In contrast, significantly reduced E-cadherin ectodomain shedding was observed during infection with the isogenic Δ*htrA* or S197A mutants (Figure 5 and data not shown). We also found that fibronectin is not cleaved during *C. jejuni* infection (not shown), which is in agreement with the earlier findings that fibronectin is a major host cell factor necessary for *C. jejuni* binding and invasion [13,16,17].

### Wild-type *C. jejuni* transmigrate efficiently across polarised MKN-28 monolayers but do not reduce TER

**Figure 6:**
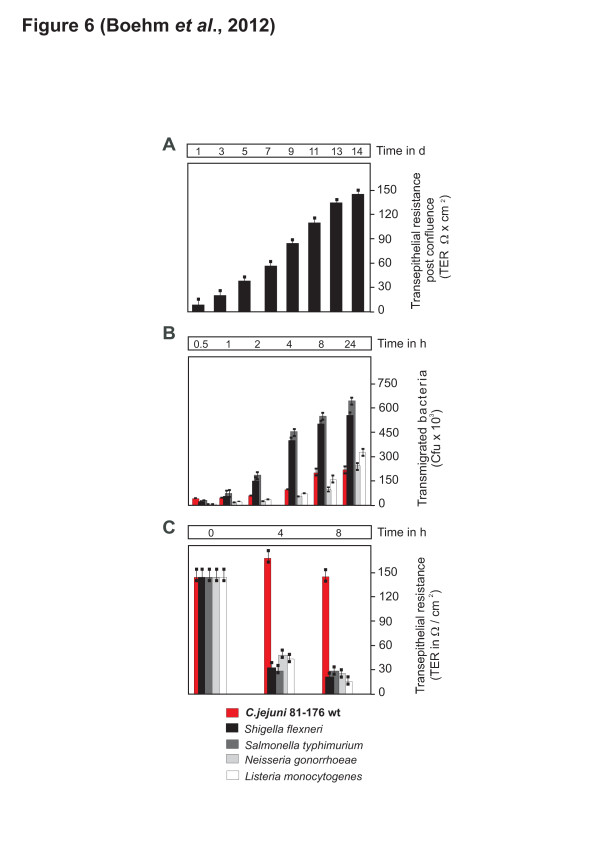
**Transmigration characteristics of different bacterial pathogens across polarised MKN-28 cells using a transwell filter system. (A)** MKN-28 cells were grown to reach monolayers in a transwell filter system. The cells were then differentiated and TER was allowed to establish over 14 days as indicated. **(B)** MKN-28 cells were then infected and CFUs of transmigrated *C. jejuni* wt strain 81–176 and some other indicated bacterial pathogens in a time course. Transmigrated bacteria were harvested from the bottom chambers, grown on MH plates, and CFUs were determined in triplicates. **(C)** TER measurement of infected MKN-28 cells during the indicated time course

Is HtrA activity important for transmigration of *C. jejuni* across polarised epithelial cells? To answer this question, MKN-28 cells were seeded and differentiated in a transwell-filter system. The transepithelial electrical resistance (TER) was followed over time and TER values between 140–150 Ω/cm^2^ were achieved 14 days after reaching confluence (Figure 6A), similarly to previously reported data [42]. Proper cell monolayers and junction formation were confirmed by E-cadherin and JAM staining in immunofluorescence microscopy [32]. MKN-28 cells were then infected with *C. jejuni* and other invasive pathogens as controls, including *Salmonella typhimurium, Shigella flexneri, Neisseria gonorrhoeae* and *Listeria monocytogenes* for 0.5 to 24 h, followed by determination of the colony forming units (CFU) in the lower chambers. The results show that *C. jejuni* transmigrated quickly, even much faster than the other pathogens during the first 30 min and increased up to 200,000 CFU over time (Figure 6B). At different time points between 2–24 h, the transmigration rates of *C. jejuni* were similar to that of *N. gonorrhoeae* and *L. monocytogenes,* but about 3 times lower than *S. typhimurium* or *S. flexneri* (Figure 6B). We also noted that *S. typhimurium* or *S. flexneri* multiply quickly in culture medium as compared to the other bacteria that did not, which can explain these higher CFUs at time points >24 h. Non-pathogenic *E. coli* Top10 did not transmigrate under the same conditions as expected (data not shown). Interestingly, the measurement of TER during infection revealed that while *S. typhimurium, S. flexneri, N. gonorrhoeae* and *L. monocytogenes* reduced TER substantially over time, infection with *C. jejuni* did not influence TER significantly (Figure 6C). This indicates that *C. jejuni,* in contrast to the other pathogens, does not decrease TER. Thus, *C. jejuni* does not induce a permanent opening of the cell-to-cell junctions in order to induce its transmigration.

### *C. jejuni* Δ*htrA* and S197A point mutants have a strong defect in transmigration

**Figure 7:**
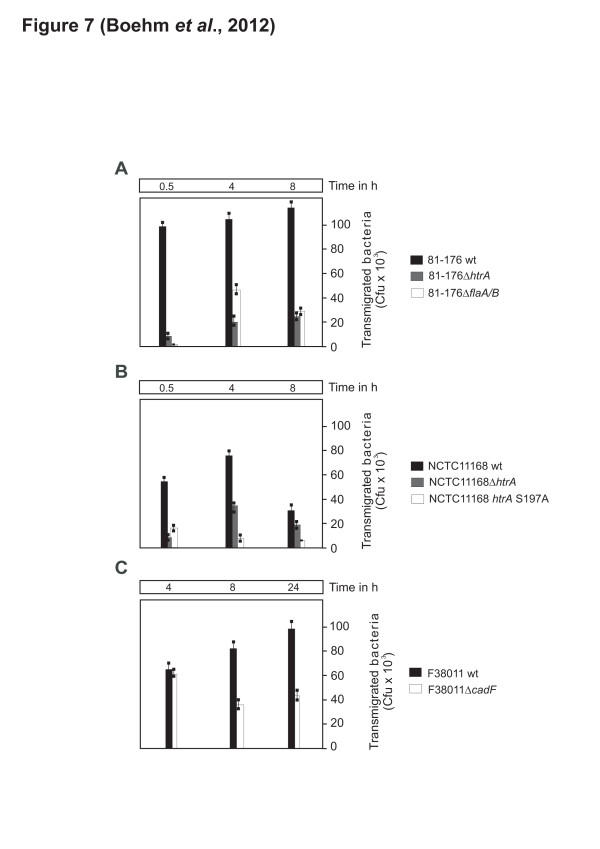
**Role of HtrA in transmigration of*C. jejuni*across polarised MKN-28 cells. (A-C)** Differentiated MKN-28 epithelial cells were grown in a transwell filter system for 14 days, and then infected with the indicated wild-type (wt) and mutant *C. jejuni* strains in the indicated time course. Transmigrated bacteria were harvested from the bottom chambers, grown on MH plates, and CFUs were determined in triplicates

To finally investigate if the expression of HtrA is important for triggering transmigration of the bacteria across a polarised epithelium, MKN-28 cells were grown and differentiated as described above, followed by infection with *C. jejuni* wt strains 81–176 or NCTC11168 and their isogenic Δ*htrA* deletion mutants. It could be shown that both Δ*htrA* mutants exhibited a strong defect in transmigration as compared to wt *C. jejuni* (Figure 7A /B). In addition, *C. jejuni* expressing the protease-inactive S197A point mutant and a flagellar mutant (Δ*flaA/B*) were also widely deficient in transmigration, while a Δ*cadF* mutant showed similar transmigration rates at the 4 h time point (Figure 7B /C). These observations suggest that secreted HtrA of *C. jejuni* and its protease activity, but also the flagellar-driven motility play crucial roles in crossing the epithelial barrier by this pathogen.

## Conclusions

The intestinal mucosa in the human intestine forms a tight barrier, which protects against host invasion by commensals, non-pathogenic microbes residing in the intestinal lumen. Some enteric pathogenic bacteria, such as *SalmonellaShigella*, or *Listeria*, have specific tissue-invading properties and can physically breach the intestinal mucosal barrier [43-45]. In general, these bacterial pathogens can translocate via a paracellular route or a transcellular route. A well studied example is *Salmonella enterica* serovar Typhimurium which can cross the intestinal barrier preferentially by entering M cells, although they can also enter and pass through epithelial cells of the intestinal tract in vivo and in cultured polarized epithelial cells in vitro [46-48]. However, very little is known about *C. jejuni* transmigration*.* Previous work has revealed that *C*. *jejuni* can translocate across Caco-2 and other polarized cell monolayers without a concomitant loss in TER [25,49-51], indicating that *C*. *jejuni* can cross a given polarised cell monolayer whose integrity, however, remains intact. In contrast, other research groups reported on a time-dependent decrease of TER caused by *C. jejuni* infection, while the bacterial factor(s) triggering a reduction in TER were not addressed [52-54]. Thus, there are some conflicting data in the literature and a consensus is yet to be reached among investigators as to the mechanism of translocation.

Our previous data suggested that HtrA chaperone activity plays a major role in *C. jejuni* host cell binding, whereas HtrA protease activity mainly affected invasion [36]. Novel data presented in this work show that HtrA from *C. jejuni* can be secreted into the cell culture supernatant during bacterial growth or during infection. In addition, it was shown that *C. jejuni* can cross polarised epithelial monolayers very rapidly. The first viable transmigrated wt *C. jejuni* CFU were detected after 15–30 min (Figure 6 and data not shown). In contrast, *C. jejuni* invasion of different host cell types was commonly observed at much later time points and was obvious between 4–6 hours or later during infection [18-21,55,56]. These facts alone already indicate that transmigration of *C. jejuni* exclude the transcellular route as a major mechanism in MKN-28 cells, which would of course take much longer time until the first bacteria reach the basolateral compartment. Instead, our findings strongly argue for the paracellular route mainly used by *C. jejuni* 81*–*176 and NCTC11168. Moreover, it was found that deletion of *htrA* or complementation with a protease-inactive S197A mutant exhibited a strongly reduced transmigration potential, indicating that HtrA’s protease activity indeed plays a role in this process. In addition, all *htrA* mutants described here expressed flagella and were highly motile. Thus, we describe here the first *C. jejuni* mutants with very high motility, but having very low transmigration and invasion potential, thus behaving like a classical avirulent Δ*flaA/B* mutant.

In addition, evidence was presented that recombinant HtrA from *C. jejuni* can cleave-off in vitro and during infection in vivo the NTF domain from E-cadherin, a major adherens junctional protein, while it leaves the receptor molecule fibronectin uncleaved. Thus, cleavage of E-cadherin may be involved in *C. jejuni* transmigration. The exact cleavage site(s) in E-cadherin, however, are yet unknown and should be investigated in future studies. In addition, the total amount of cell-based E-cadherin dropped down during the course of infection, but did not lead to a complete cleavage, even at late time points of infection (8 hours). We therefore propose that cleavage of E-cadherin by HtrA during infection is a strictly controlled, temporary and locally restricted process, possibly achieved by surface-exposed and/or secreted HtrA proteins when the bacteria enter the intercellular space. Host cells continuously translate large amounts of E-cadherin proteins, and therefore the host cell machinery could rapidly replace cleaved proteins. This hypothesis could also explain why no significant reduction in TER was observed during infection with *C. jejuni*, and suggests that these bacteria can close the “door” behind them, which appears as a clever novel infection mechanism during bacterial transmigration across polarised gut epithelial cells.

## Methods

### *Campylobacter* strains

The *C. jejuni* strains RM1221, ATCC43430, TGH-9011, NCTC11168, 1849, 81–176, 1543/01, 2703/01, ST3046 and F38011 were used in this study. The isogenic mutants 81-176Δ*cadF*, 11168Δ*htrA* and 11168*htrA*S197A were recently described [33,35-37]. The isogenic F38011Δ*cadF* and 81-176Δ*flaA/B* mutants were kindly provided by Michael Konkel [57] and Patricia Guerry [58]. All *C. jejuni* strains were grown on *Campylobacter* blood-free selective Agar Base (Oxoid) containing *Campylobacter* growth supplement (Oxoid) or on Mueller-Hinton (MH) agar amended with 50 μg/ml kanamycin or 30 μg/ml or chloramphenicol at 37°C under microaerobic conditions (generated by CampyGen, Oxoid) for 48 hours.

### Other bacterial species

*Salmonella typhimurium* strain NCTC12023 was kindly provided by M. Hensel (University Osnabrueck/Germany). *Neisseria gonorrhoeae* strain 6B10 is a gift of T. Meyer (Max Planck Institute for Infection Biology Berlin/Germany). *Shigella flexneri* strain 15.4 is a clinical isolate from the Medical School Magdeburg/Germany*,* and *Listeria monocytogenes* strain EGD (Serotyp 1/2a) was kindly provided by J. Wehland (HZI Braunschweig/Germany). As control, we used the non-pathogenic *Escherichia coli* strain Top10 (Invitrogen). Each of these bacteria was grown overnight at 37°C on conventional LB agar plates.

### HtrA secretion assays

*C. jejuni* wild-type and *ΔhtrA* deletion mutant strains were grown in BHI broth medium for 12 hours to an OD_600nm_ ~1.0. The supernatant and the cell pellets were separated by centrifugation at 4,000 rpm, and the supernatant was further purified from remaining bacterial cells by passage through a 0.21 μm sterile filter. The resulting bacterial pellets and supernatants were analysed by immunoblot and casein zymography analyses. Absence of live bacteria in the supernatant was confirmed by incubation on agar plates showing no growth.

### Motility assays

Motility phenotypes of strains were tested in MH media containing 0.4% agar. Bacterial cells were harvested from a 36 h culture on conventional agar plates and resuspended in PBS to obtain an optical density at 600 nm of 0.45 (approximately 1 × 10^9^ CFU/ml). Subsequently, 2 μl of a bacterial suspension of 2 × 10^8^ CFU/ml were stabbed into motility agar. Plates were incubated at 37°C under microaerophilic conditions for 36 h, followed by measuring the diameter of the resulting swarms. The final data were the mean of at least five separate measurements from three experiments.

### Host cell lines

Human embryonic intestinal epithelial cells (INT-407, non-polarised), obtained from the American Type Culture Collection (ATCC CCL-6) and polarised MKN-28 cells were grown in RPMI-1640 medium containing L-glutamine and Earle’s salts (Gibco). After reaching about 70% confluency, the cells were washed two times with PBS, and then starved for 12 h before infection.

### Infection studies

For the infection experiments, INT-407 cells were seeded to give 4 × 10^5^ CFU in 12-well tissue culture plates. The culture medium was replaced with fresh medium without antibiotics 1 h before infection. Bacteria were suspended in culture medium, added to the cells at a multiplicity of infection (MOI) of 100, and co-incubated with host cells for the indicated periods of time per experiment.

### Transepithelial electrical resistance (TER) assay

MKN-28 cells were cultured on 0.33 cm² cell culture inserts with 3 μm pore size (Millipore). The cells were allowed to form confluent monolayers, and then incubated for another 14 days. TER was measured with an Electrical Resistance System (ERS) (Millipore). Maximum resistance indicated that the cells reached maximal polarity. TER was calculated as Ohms x cm² by subtracting fluid resistance and multiplying by the monolayer surface area. Bacteria were suspended in culture medium, added to the cells at a multiplicity of infection (MOI) of 50, and co-incubated with host cells for the indicated periods of time per experiment. The number of CFU was determined by growth on MH or LB plates, respectively.

### HtrA expression, purification and E-cadherin cleavage in vitro

Cloning of *H. pylori htrA* (*Hp*HtrA aa18-aa475) and *C. jejuni htrA* (CjHtrA aa17-aa472) was described previously [33,34]. Briefly, the genes were amplified from genomic DNA excluding predicted signal peptides. PCR fragments flanked by restriction sites for *Bam*HI/*Xma*I were cloned into pGEX-6P-1 (GE Healthcare) to generate a GST-fusion protein. The expression and purification protocol was described in detail [34]. Cleavage assays of purified HtrA with recombinant human full-length E-cadherin (R&D Systems), recombinant human His-tagged N-terminal NTF domain (Sino Biological) or human fibronectin (Calbiochem) were performed as described [32].

### SDS-PAGE and western blot

Cells were lysed [32], proteins were separated by SDS-PAGE and tested for fibronectin (Santa Cruz) and E-cadherin using polyclonal antibodies recognizing the extracellular domain of E-cadherin (H-108 from Santa Cruz or HECD1 from BD Biosciences) and whole cell lysates were tested for GAPDH. The polyclonal anti-His tag antibody is from Qiagen and the rabbit HtrA antibody was described in [36,37]. Bacterial HtrAs were detected by Coomassie staining (BioRad).

### Casein zymography

Bacterial lysates, culture supernatants or recombinant HtrA were separated in casein containing gels under non-reducing conditions. Subsequently, gels were renatured in 2.5% Triton-X-100 and equilibrated in developing buffer [34]. Caseinolytic activity was visualized by staining with 0.5% Coomassie Blue R250.

### Field emission scanning electron microscopy (FESEM)

Plate-grown *C. jejuni* strains were harvested and fixed in a sterile solution containing 5% formaldehyde, 2% glutaraldehyde in cacodylate buffer (0.1 M cacodylate, 0.01 M CaCl_2_, 0.01 M MgCl_2_, 0.09 M sucrose, pH 6.9) for 1 hour on ice. The solution was centrifuged and passed through a sterile filter. After several washes with cacodylate buffer and TE buffer (20 mM Tris, 1 mM EDTA, pH 6.9), samples were dehydrated in serial dilutions of acetone (10, 30, 50, 70, 90 and 100%) on ice for 15 min each step. Samples were then allowed to reach room temperature before another change of 100% acetone, after which they were subjected to critical-point drying with liquid CO_2_ (CPD030, Bal-Tec). Samples were finally covered with ca. 10.0-nm 11 thick gold film by sputter coating (SCD500, Bal-Tec) and examined in a field emission scanning electron microscope (Zeiss DSM 982 Gemini) using an Everhart Thornley SE detector and in-lens detector in a 50:50 ratio at an acceleration voltage of 5.0 kV.

### Electron microscopic analysis by negative staining

For negative staining, thin carbon support films were prepared by indirect sublimation of carbon on freshly cleaved mica. Samples were then absorbed to the carbon film and negatively stained with 1% (wt/vol) aqueous uranyl acetate (pH 4.5). After air drying, samples were examined by transmission electron microscopy (TEM) in a Zeiss TEM 910 at an acceleration voltage of 80 kV.

### Statistical analysis

All data were evaluated using Student *t*-test with SigmaStat statistical software (version 2.0). Statistical significance was defined by *P* ≤ 0.05 (*) and *P* ≤ 0.005 (**). All error bars shown in figures and those quoted following the +/− signs represent standard deviations.

## Competing interests

The authors declare that they have no competing interests.

## Authors’ contributions

MB, BH, MR and NT performed and designed the experiments. KTB, LB, OAO and SW provided crucial materials and advise for the experiments. SB, the senior/corresponding author, supervised the experiments and wrote the manuscript together with SW. All authors read and approved the final manuscript.

## Supplementary Material

Additional file 1 **Figure S1Structural and sequence comparison of*C. jejuni*and*H. pylori*HtrA proteins. (A)** Schematic diagram of the domain arrangement of HtrAs from *C. jejuni* (*Cj*) and *H. pylori* (*Hp*). **(B)** Multiple sequence alignment of HtrA from different *C. jejuni* and *H. pylori* strains. The protein sequences of *Cj* 81*–*176, *Cj* RM1221, *Cj* NCTC11168, *Hp* 26695, *Hp* P12 and *Hp* 35A are aligned. The conserved amino acids of the catalytic triad are indicated in red and shaded with yellow (H: Histidine; D: Aspartic acid; S: Serine). **Figure S2. Analysis of wild-type*C. jejuni*and*htrA*mutants by negative staining and electron microscopy.** Investigation revealed that wild-type (wt) *C. jejuni* and different *htrA* mutants of strain 81–176 and NCTC11168 produce intact bipolar flagella (blue arrows) and only slight morphological differences, thus confirming results from FESEM (Figure 1). Each bar corresponds to 1 μm. **Figure S3. Overexpression and purification of*C. jejuni*HtrA.***C. jejuni* HtrA was expressed as GST-tag fusion in *E. coli* BL-21, and then purified as described in Materials and Methods. A Coomassie-stained gel of different fractions and purified HtrA proteins during the purification procedure is shown. **Figure S4. Recombinant*C. jejuni*HtrA cleaves E-cadherin but not fibronectin at 42**°C**.** The experiments were performed under identical conditions as shown in Figure 4D /E, but were incubated not incubated at 37°C but 42°C. **(A)** In vitro cleavage of the recombinant E-cadherin NTF domain performed with purified *Cj* HtrA or *Hp* HtrA results in several indicated subfragments. **(B)** In vitro cleavage assay of fibronectin incubated with purified HtrAs under identical conditions shows that fibronectin can be cleaved by *Hp* HtrA, but not *Cj* HtrA. All reactions were incubated for 16 h. Click here for file
