# Supplementary material for: Rapid paracellular transmigration of Campylobacter jejuni across polarized epithelial cells without affecting TER: role of proteolytic-active HtrA cleaving E-cadherin but not fibronectin
Source: Gut Pathog. 2012 Apr 25;4:3. doi: 10.1186/1757-4749-4-3 (PMC3413534; doi:10.1186/1757-4749-4-3)

## Supplemental materials:

**Additional Figure 1: Structural and sequence comparison of *C. jejuni* and *H. pylori* HtrA proteins.** (A) Schematic diagram of the domain arrangement of HtrAs from *C. jejuni* (*Cj*) and *H. pylori* (*Hp*). (B) Multiple sequence alignment of HtrA from different *C. jejuni* and *H. pylori* strains. The protein sequences of *Cj* 81-176, *Cj* RM1221, *Cj* NCTC11168, *Hp* 26695, *Hp* P12 and *Hp* 35A are aligned. The conserved amino acids of the catalytic triad are indicated in red and shaded with yellow (H: Histidine; D: Aspartic acid; S: Serine).

**Additional Figure 2: Analysis of wild-type *C. jejuni* and *htrA* mutants by negative staining and electron microscopy.** Investigation revealed that wild-type (wt) *C. jejuni* and different *htrA* mutants of strain 81-176 and NCTC11168 produce intact bipolar flagella (blue arrows) and only slight morphological differences, thus confirming results from FESEM (Figure 1). Each bar corresponds to 1µm.

**Additional Figure 3: Overexpression and purification of *C. jejuni* HtrA.** *C. jejuni* HtrA was expressed as GST-tag fusion in *E. coli* BL-21, and then purified as described in Materials and Methods. A Coomassie-stained gel of different fractions and purified HtrA proteins during the purification procedure is shown.

**Additional Figure 4: Recombinant *C. jejuni* HtrA cleaves E-cadherin but not fibronectin at 42°C.** The experiments were performed under identical conditions as shown in Figure 4D/E, but were incubated not incubated at 37°C but 42°C. (A) *In vitro* cleavage of the recombinant E-cadherin NTF domain performed with purified *Cj* HtrA or *Hp* HtrA results in several indicated subfragments. (B) *In vitro* cleavage assay of fibronectin incubated with purified HtrAs under identical conditions shows that fibronectin can be cleaved by *Hp* HtrA, but not *Cj* HtrA. All reactions were incubated for 16 h.

# Additional Figure S1 (Boehm *et al.*, 2012)

**A**

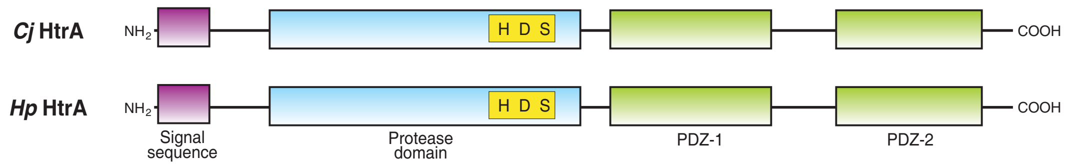


**B**

```
Cj81-176        -MKK-IFLSLSLASALFAASINFNESTATANRVN-PAAGNAVLSYHDSIKDAKKSVVNIST 58
CjRM1221        -MKK-IFLSLSLASALFAASINFNESTATANRVN-PAAGNAVLSYHDSIKDAKKSVVNIST 58
CjNCTC11168     -MKK-IFLSLSLASALFAASINFNESTATANRVN-PAAGNAVLSYHDSIKDAKKSVVNIST 58
Hp26695         MMKKTLFISLALALSLNAGNIQIQSMPKVKERVSVPSKDDTIYSYHDSIKDSIKAVVNIST 61
HpP12           MMKKTLFISLALALSLNAGNIQIQSMPKVKERVSVPSKDDTIYSYHDSIKDSIKAVVNIST 61
Hp35A           MMKKTLFISLALALSLNAGNIQIQSMPKVKERVSVPSKDDTIYSYHDSIKDSIKAVVNIST 61
                 *** :*:**:** :* *..*:::. . . :**. *: .::: ********: *:******

Cj81-176        SKTITRANRPSPLDDFFNDPYFKQFFDFDFPQRKGKNDKEVVSSLGSGVIISKDGYIVTNN 119
CjRM1221        SKTITRANRPSPLDDFFNDPYFKQFFDFDFPQRKGKNDKEVVSSLGSGVIISKDGYIVTNN 119
CjNCTC11168     SKTITRANRPSPLDDFFNDPYFKQFFDFDFSQRKGKNDKEVVSSLGSGVIISKDGYIVTNN 119
Hp26695         EKKIKNNFIGG---GVFNDPFFQQFFG-DLGGMIPK--ERMERALGSGVIISKDGYIVTNN 116
HpP12           EKKIKNNFIGG---GVFNDPFFQQFFG-DLGGMIPK--ERMERALGSGVIISKDGYIVTNN 116
Hp35A           EKKIKNNFIGG---GVFNDPFFQQFFG-DLGGMIPK--ERMERALGSGVIISKDGYIVTNN 116
                .*.*..    .   ..****:*:***. *:      *  :.:  :****************

Cj81-176        HVVDDADTITVNLPGSDIEYKAKLIGKDPKTDLAVIKIEANNLSAITFTNSDDLMEGDVVF 180
CjRM1221        HVVDDADTITVNLPGSDIEYKAKLIGKDPKTDLAVIKIEANNLSAITFTNSDDLMEGDVVF 180
CjNCTC11168     HVVDDADTITVNLPGSDIEYKAKLIGKDPKTDLAVIKIEANNLSAITFTNSDDLMEGDVVF 180
Hp26695         HVIDGADKIKVTIPGSNKEYSATLVGTDSESDLAVIRITKDNLPTIKFSDSNDISVGDLVF 177
HpP12           HVIDGADKIKVTIPGSNKEYSATLVGTDSESDLAVIRITKDNLPTIKFSDSNDISVGDLVF 177
Hp35A           HVIDGADKIKVTIPGSNKEYSATLVGTDSESDLAVIRITKDNLPTIKFSDSNDILVGDLVF 177
                **:*.**.*.*.:***: **.*.*:*.*.::*****:*  :**.:*.*::*:*:  **:**

Cj81-176        ALGNPFGVGFSVTSGIISALNKDNIGLNQYENFIQTDASINPGNSGGALVDSRGYLVGINS 241
CjRM1221        ALGNPFGVGFSVTSGIISALNKDNIGLNQYENFIQTDASINPGNSGGALVDSRGYLVGINS 241
CjNCTC11168     ALGNPFGVGFSVTSGIISALNKDNIGLNQYENFIQTDASINPGNSGGALVDSRGYLVGINS 241
Hp26695         AIGNPFGVGESVTQGIVSALNKSGIGINSYENFIQTDASINPGNSGGALIDSRGGLVGINT 238
HpP12           AIGNPFGVGESVTQGIVSALNKSGIGINSYENFIQTDASINPGNSGGALIDSRGGLVGINT 238
Hp35A           AIGNPFGVGESVTQGIVSALNKSGIGINSYENFIQTDASINPGNSGGALIDSRGGLVGINT 238
                *:******* ***.**:*****..**:*.*******************:**** *****:

Cj81-176        AILSRGGGNNGIGFAIPSNMVKDIAKKLIEKGKIDRGFLGVTILALQGDTKKAYKNQEGAL 302
CjRM1221        AILSRGGGNNGIGFAIPSNMVKDIAKKLIEKGKIDRGFLGVTILALQGDTKKAYKNQEGAL 302
CjNCTC11168     AILSRGGGNNGIGFAIPSNMVKDIAKKLIEKGKIDRGFLGVTILALQGDTKKAYKNQEGAL 302
Hp26695         AIISKTGGNHGIGFAIPSNMVKDTVTQLIKTGKIERGYLGVGLQDLSGDLQNSYDNKEGAV 299
HpP12           AIISKTGGNHGIGFAIPSNMVKDIVTQLIKTGKIERGYLGVGLQDLSGDLQNSYDNKEGAV 299
Hp35A           AIISKTGGNHGIGFAIPSNMVKDIVTQLIKTGKIERGYLGVGLQDLSGDLQNSYDNKEGAV 299
                **:*: ***:************* ..:**:.***:**:*** :  *.** :::*.*:***:

Cj81-176        ITDVQKGSSADEAGLKRGDLVTKVNNKVIKSPIDLKNYIGTLEIGQKISLSYERDGENKQA 362
CjRM1221        ITDVQKGSSADEAGLKRGDLVTKVNDKVIKSPIDLKNYIGTLEIGQKISLSYERDGENKQA 362
CjNCTC11168     ITDVQKGSSADEAGLKRGDLVTKVNDKVIKSPIDLKNYIGTLEIGQKISLSYERDGENKQA 362
Hp26695         VISVEKDSPAKKAGILVWDLITEVNGKKVKNTNELRNLIGSMLPNQRVTLKVIRDKKERAF 359
HpP12           VISVEKDSPAKKAGILVWDLITEVNGKKVKNTNELRNLIGSMLPNQRVTLKVIRDKKERAF 359
Hp35A           VISVEKDSPAKKVGILVWDLITEVNGKKVKNTNELRNLIGSMLPNQRVTLKVIRDKKERTF 359
                : .*:*.*.*.:.*:   **:*:**.* :*.. :*:* **:: .*:::*.  ** :::

Cj81-176        SFILKGEKE-NPKGVQSD------LIDGLSLRNLDPRLKDRLQIPKDVNGVLVDSVKEKSK 417
CjRM1221        SFILKGEKE-NPKGVQSD------LIDGLSLRNLDPRLKDRLQIPKDVNGVLVDSVKEKSK 417
CjNCTC11168     SFILKGEKE-NPKGVQSD------LIDGLSLRNLDPRLKDRLQIPKDVNGVLVDSVKEKSK 417
Hp26695         TLTLAERKNPNKKETISAQNGAQGQLNGLQVEDLTQETKRSMRLSDDVQGVLVSQVNENSP 421
HpP12           TLTLAERKNPNKKETISAQNGAQGQLNGLQVEDLTQKTKRSMRLSDDVQGVLVSQVNENSP 421
Hp35A           TLTLAERKNPNKKETISAQNGSQGQLNGLQVEDLTQKTKRSMRLSDDVQGVLVSQVNENSP 421
                :: *  .*: * * . *        ::**.:.:*  . *  :::..**:****. .*:*:*

Cj81-176        GKNSGFQEGDIIIGVGQSEIKNLKDLEQALKQVN-KKEFTKVWVYRNGFATLLVLK 472
CjRM1221        GKNSGFQEGDIIIGVGQSEIKNLKDLEQALKQVN-KKEFTKVWVYRNGFATLLVLK 472
CjNCTC11168     GKNSGFQEGDIIIGVGQSEIKNLKDLEQALKQVN-KKEFTKVWVYRNGFATLLVLK 472
Hp26695         AEQAGFRQGNIITKIEEVEVKSVADFNHALEKYKGKPKRFLVLDLNQGYRIILVK- 476
HpP12           AEQAGFRQGNIITKIEEVEVKSVADFNHALEKYKGKPKRFLVLDLNQGYRIILVK- 476
Hp35A           AEQAGFRQGNIITKIEEVEVKSIADFNHALEKYKGKPKRFLVLDLNQGYRIVLVK- 476
                .:::**::*:** : : *:*.: *:::**:: : * :    *   .:*:  :**
```

# Additional Figure S2 (Boehm *et al.*, 2012)

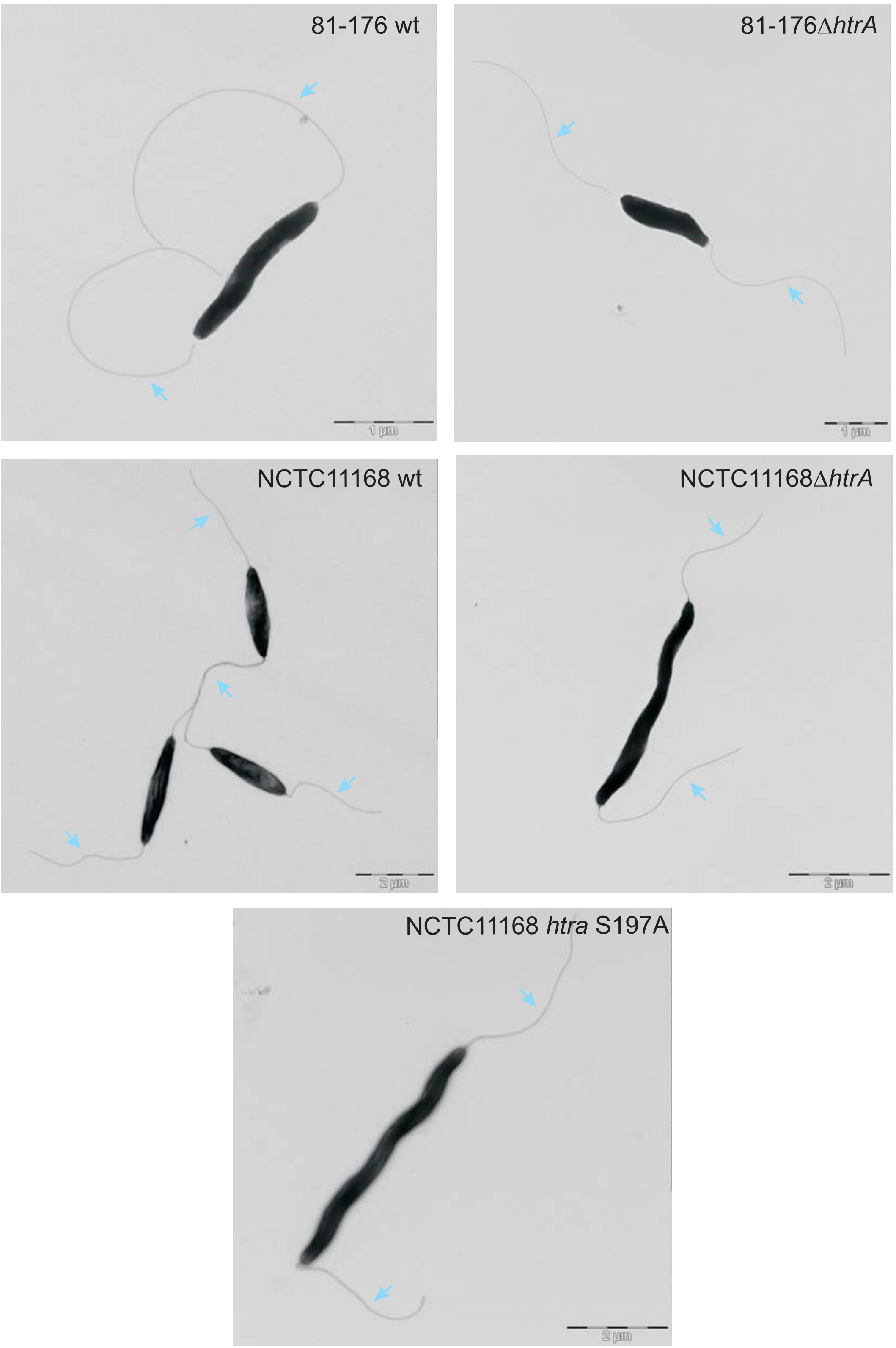

# Additional Figure S3 (Boehm *et al.*, 2012)

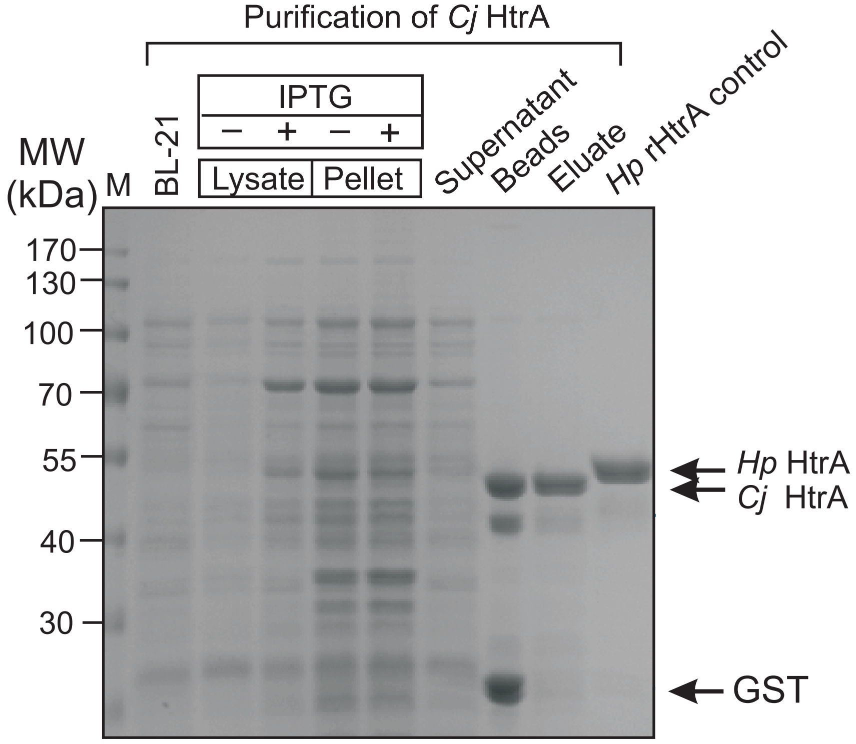

# Additional Figure S4 (Boehm *et al.*, 2012)

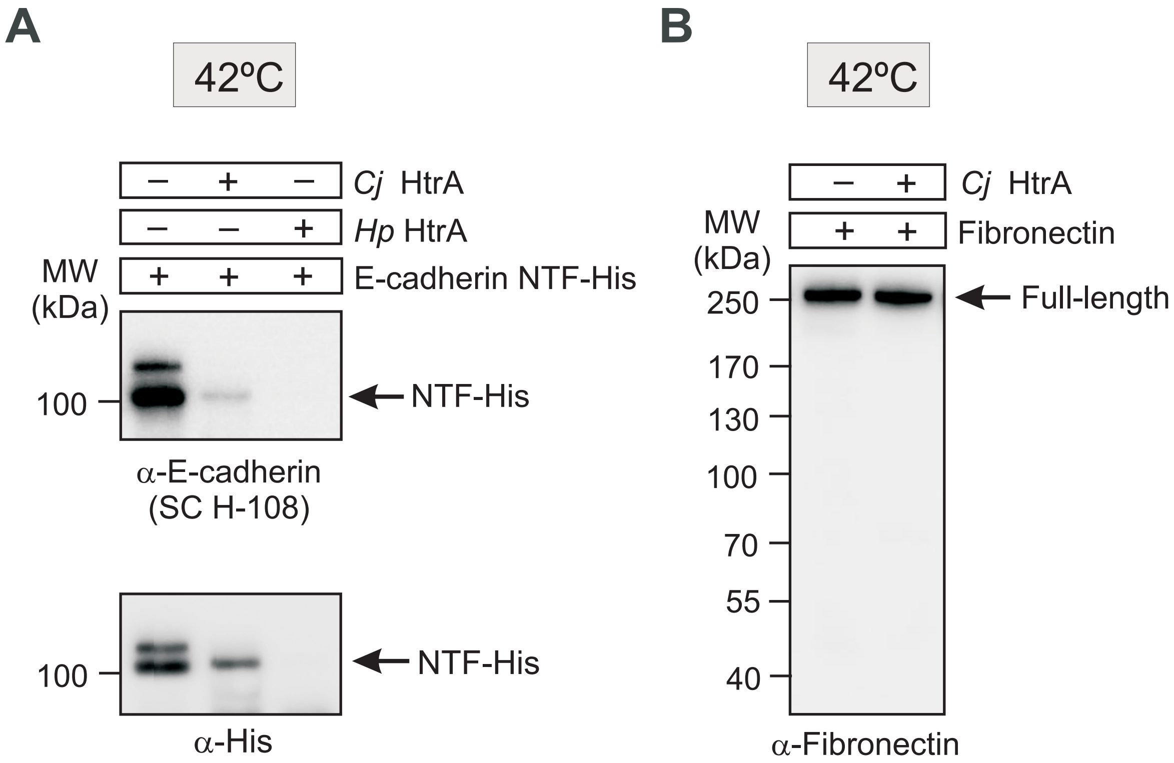

Supplement: Additional file 1 — Figure S1Structural and sequence comparison ofC. jejuniandH. pyloriHtrA proteins. (A) Schematic diagram of the domain arrangement of HtrAs from C. jejuni (Cj) and H. pylori (Hp). (B) Multiple sequence alignment of HtrA from different C. jejuni and H. pylori strains. The protein sequences of Cj 81–176, Cj RM1221, Cj NCTC11168, Hp 26695, Hp P12 and Hp 35A are aligned. The conserved amino acids of the catalytic triad are indicated in red and shaded with yellow (H: Histidine; D: Aspartic acid; S: Serine). Figure S2. Analysis of wild-typeC. jejuniandhtrAmutants by negative staining and electron microscopy. Investigation revealed that wild-type (wt) C. jejuni and different htrA mutants of strain 81–176 and NCTC11168 produce intact bipolar flagella (blue arrows) and only slight morphological differences, thus confirming results from FESEM (Figure 1). Each bar corresponds to 1 μm. Figure S3. Overexpression and purification ofC. jejuniHtrA.C. jejuni HtrA was expressed as GST-tag fusion in E. coli BL-21, and then purified as described in Materials and Methods. A Coomassie-stained gel of different fractions and purified HtrA proteins during the purification procedure is shown. Figure S4. RecombinantC. jejuniHtrA cleaves E-cadherin but not fibronectin at 42°C. The experiments were performed under identical conditions as shown in Figure 4D /E, but were incubated not incubated at 37°C but 42°C. (A) In vitro cleavage of the recombinant E-cadherin NTF domain performed with purified Cj HtrA or Hp HtrA results in several indicated subfragments. (B) In vitro cleavage assay of fibronectin incubated with purified HtrAs under identical conditions shows that fibronectin can be cleaved by Hp HtrA, but not Cj HtrA. All reactions were incubated for 16 h. [file 1757-4749-4-3-S1.pdf]
